# Prenatal loss of control eating is associated with psychiatric symptoms and distress among individuals with elevated BMI

**DOI:** 10.1080/0167482X.2025.2483283

**Published:** 2025-04-02

**Authors:** Michele D. Levine, Riley J. Jouppi, Rachel P. Kolko Conlon, Christine C. Call, Jennifer L. Grace, Gina M. Sweeny, Zijing Zhang

**Affiliations:** a Department of Psychiatry, University of Pittsburgh Medical Center, Pittsburgh, PA, USA; b Department of Psychology, University of Pittsburgh, Pittsburgh, PA, USA

**Keywords:** Pregnancy, loss of control eating, binge eating, depression, body image

## Abstract

**Purpose:**

Loss of control while eating (LOC) or feeling unable to control the amount or type of food consumed during an eating episode, is the core psychopathology in binge eating disorders. Yet, the impact of LOC on other psychiatric symptoms during pregnancy is not known. This study evaluated the contribution of prenatal LOC to psychological distress and disordered eating attitudes.

**Methods:**

Pregnant individuals with BMI ≥ 25 (*N* = 312) recruited for a perinatal health promotion trial self-reported past-month LOC; eating, shape, and weight concerns; prenatal depressive symptoms, anxiety, and stress. Propensity scores were used to reduce bias associated with cross-sectional data.

**Results:**

Overall, 34.3% (*n* = 107) reported LOC. Individuals with prenatal LOC, relative to those without, endorsed more eating disorder symptoms (*p*s<.001) as well as more symptoms of depression (7.1±0.3 vs. 5.4±4.9) and anxiety (38.1 ± 11.7 vs. 33.4 ± 11.7) and greater perceived stress (25.0 ± 7.9 vs. 22.0±9.9, *p*s<.001). LOC frequency was associated with significantly more prenatal psychological distress, beyond the effect of other factors that increase the likelihood of LOC. *(ps*<.005).

**Conclusions:**

Among individuals with elevated BMI, prenatal LOC is common and relates to eating disorder and other psychiatric symptoms. Prenatal LOC may represent a behavioral mechanism for improved psychological health.

## Introduction

Loss of control eating (LOC) refers to the ingestion of food while feeling unable to control the amount or type of food eaten and is the fundamental element of binge eating disorders [1,2]. Indeed, there is consensus that the experience of LOC, regardless of the amount or type of food consumed, is the aspect of binge eating consistently associated with clinical impairment and distress [3]. Importantly, while LOC represents the core psychopathology in diagnosable binge eating disorders, including bulimia nervosa and binge eating disorder, LOC has also been reported in samples without diagnosed eating disorders [4]. For example, as many as 40% of adolescents and young adults report LOC (e.g. in adults, approximately 30% of individuals who present for weight management interventions meet criteria for binge eating [5,6]), and as many as 77% of adult veterans presenting for weight management services through the U.S. Department of Veterans Affairs report LOC [7]. LOC also is more prevalent among individuals with higher body mass index (BMI) [8,9]. Moreover, relative to individuals who do not experience LOC, those who do report more frequent eating episodes, are more likely to consume palatable foods in response to stress, and experience greater reinforcement from eating palatable foods in behavioral paradigms and neuroimaging studies [10,11]. LOC has been linked to increased inflammation and cortisol levels as well as overall down-regulation of the hypothalamic-pituitary-adrenal axis [12] and thus represents an important health marker.

LOC also is the most frequently reported disordered eating behavior during pregnancy [13–15]. Our research [16], and that of others [17,18], documents that as many as 41% of individuals with BMIs ≥25 report LOC during the perinatal period. In addition, although other eating disorder symptoms often decrease during pregnancy [17,19], LOC persists and may even develop during pregnancy. For example, among individuals with BMI ≥25, rates of LOC are higher during pregnancy than in the period prior to pregnancy or in the postpartum period [16]. Evidence further suggests that the experience of LOC during pregnancy is associated with increased gestational weight gain in general [20,21], and may be uniquely predictive of gestational weight gain among individuals with elevated BMI who are at high risk for excessive gestation weight gain [22]. Prenatal LOC also has been linked to poorer cardiovascular health [23]. Given that prenatal LOC is common and associated with negative health outcomes, understanding the relationship between LOC and other markers of psychological well-being can inform efforts to address prenatal LOC.

Outside of the context of pregnancy, the experience of LOC has been robustly linked to psychological distress, including depressive symptoms, anxiety, general psychopathology, and disordered eating attitudes such as increased concerns about body shape and weight [1,2,4,24]. Initial evidence suggests that LOC during pregnancy is similarly associated with markers of psychological distress. For example, our previous work has shown prenatal LOC to be associated with depressive symptoms and perceived stress among pregnant individuals with elevated BMI [25]. Other research has similarly reported an association between prenatal disordered eating behavior and depressive symptoms, among individuals with a pre-pregnancy history of an eating disorder [25,26]. However, the relationship between prenatal LOC and related disordered eating attitudes is not clear. Additionally, given that outside of pregnancy, higher rates of LOC have been documented among individuals with a BMI ≥25, research designed to replicate and extend findings related to the associations between prenatal LOC and other measures of psychological distress as well as with eating disorder attitudes among those with elevated BMIs is needed.

Accordingly, the present study sought to examine the contribution of past-month, self-reported LOC to prenatal psychological distress and disordered eating attitudes among pregnant individuals with BMIs ≥25 enrolled in an ongoing trial to optimize health and well-being at one year postpartum. We hypothesized that LOC during pregnancy would be associated with more depressive and anxiety symptoms and greater perceived stress, as well as with more concerns about eating, shape, and weight. We expected that these differences would persist after adjusting for confounds that may relate to both the likelihood of experiencing LOC and other markers of general and eating disorder psychopathology. We further expected that greater frequency of LOC episodes would relate to more severe psychological distress and disordered eating psychopathology.

## Methods

### Participants and procedures

Pregnant individuals (*N* = 312) were recruited from obstetric clinics associated with a large urban hospital to participate in a behavioral trial designed to address perinatal health and wellbeing [27]. Individuals were eligible to participate if they had a pre-pregnancy body mass index (BMI)≥25kg/m^2^, were 12–20 weeks’ gestation, were ≥14 years old, and had a singleton pregnancy. Exclusion criteria were use of weight-affecting medications (e.g. steroids) or medical conditions (e.g. thyroid disorders), participation in weight-management programming, type 1 diabetes, or psychiatric symptoms requiring immediate treatment. Study procedures were approved by the local Institutional Review Board, and all participants received monetary compensation for participation.

Between December 2016 and September 2022, a total of 1,373 individuals were contacted about the study and 1,051 were screened for eligibility. Of the 409 determined to be interested and eligible, 320 individuals attended a baseline assessment. At this visit, written consent was obtained, and baseline self-report measures were collected. A total of 312 participants completed their baseline assessment and were eligible to continue the study. For this secondary data analysis, all data were drawn from the baseline visit prior to randomization.

### Assessments

#### Demographic and clinical information

Participants self-reported demographic information, including age, racial and ethnic identities, annual household income, educational background, and parity. Individuals were asked to describe their ethnic identity as either Hispanic or Latina or not and to circle all categories that described their racial identity. Participants also were queried about tobacco smoking, and smoking status was coded as ever relative to never.

#### Pre-pregnancy weight

During the initial phone screen, participants self-reported pre-pregnancy weight, a commonly used and valid method of obtaining pre-pregnancy weight [28]. During the assessment, height and weight were measured in-person *via* a calibrated stadiometer and digital scale, respectively. Pre-pregnancy BMI was calculated using self-reported pre-pregnancy weight obtained during the initial phone screen and measured height at the assessment.

#### Eating disorder examination–questionnaire (EDE-Q)

The EDE-Q is a 28-item self-report measure designed to assess eating disorder psychopathology and associated features. Questions are answered from 0 (“Never”) to 6 (“Every day”), and higher scores indicate greater eating disorder psychopathology [29]. The EDE-Q produces three subscale scores corresponding to eating disorder attitudes, including *Weight Concern*, *Shape Concern*, and *Eating Concern*. The EDE-Q also prompts for the frequency of LOC eating episodes. We defined LOC as the self-report of any experience of loss of control during the past 28 days. Responses to questions about the number of times LOC was present during eating episodes when eating an unusually large amount of food were used to create the dichotomized LOC present or absent variable. In addition, the EDE-Q prompts for self-report of the number episodes of LOC reported in past 28 days.

#### Psychological distress

Participants self-reported depressive symptoms over the past week on the Edinburgh Postnatal Depression Scale (EPDS) [30]. State anxiety was assessed using the State-Trait Anxiety Inventory (STAI) [31]. Participants also completed the Perceived Stress Scale (PSS) [32]. The STAI and PSS have been administered among pregnant people and demonstrated adequate psychometric properties [33,34].

### Analytic plan

Descriptive statistics were calculated to characterize the sample and are shown in Table 1. Analyses were designed to first test the association of past-month prenatal LOC to psychological distress and eating, shape, and weight concerns during early pregnancy. Second, we examined the relationship between number of self-reported episodes of LOC and psychological distress, eating, shape and weight concerns.

Because the current study was cross-sectional, and participants with and without prenatal LOC may differ in ways that affect psychological distress and/or disordered eating attitudes, we adjusted for propensity scores in all analyses to help reduce overt bias [35,36]. Propensity scores, representing risk for prenatal LOC relative to others in the sample, were estimated by logistic regression of past-month prenatal LOC on age, racial and ethnic identities, educational background, income, pre-pregnancy BMI, and smoking status, which have been linked to our variables of interest [35,37,38]. While it is not necessary to include confounders used to calculate propensity scores as additional covariates in final models since their confounding is already adjusted for through the propensity scores, doing so is considered a doubly robust method and recommended because it can compensate for insufficient covariate balance [36,39]. Thus, we included the variables indicated in Table 1 in the computation of a propensity score [36,39].

## Results

Demographic and clinical characteristics are described in Table 1. At 13.6 ± 2.7 weeks’ gestation, 34.3% of participants (*n* = 107) reported LOC. Among those who endorsed any past-month LOC (*n* = 107), the number of total LOC episodes ranged from 1 to 20, with a mean of 4.7 ± 4.5 episodes. There were no differences in sociodemographic characteristics between pregnant individuals with and without LOC.

### Prenatal LOC and eating disorder attitudes

**Figure 1.**
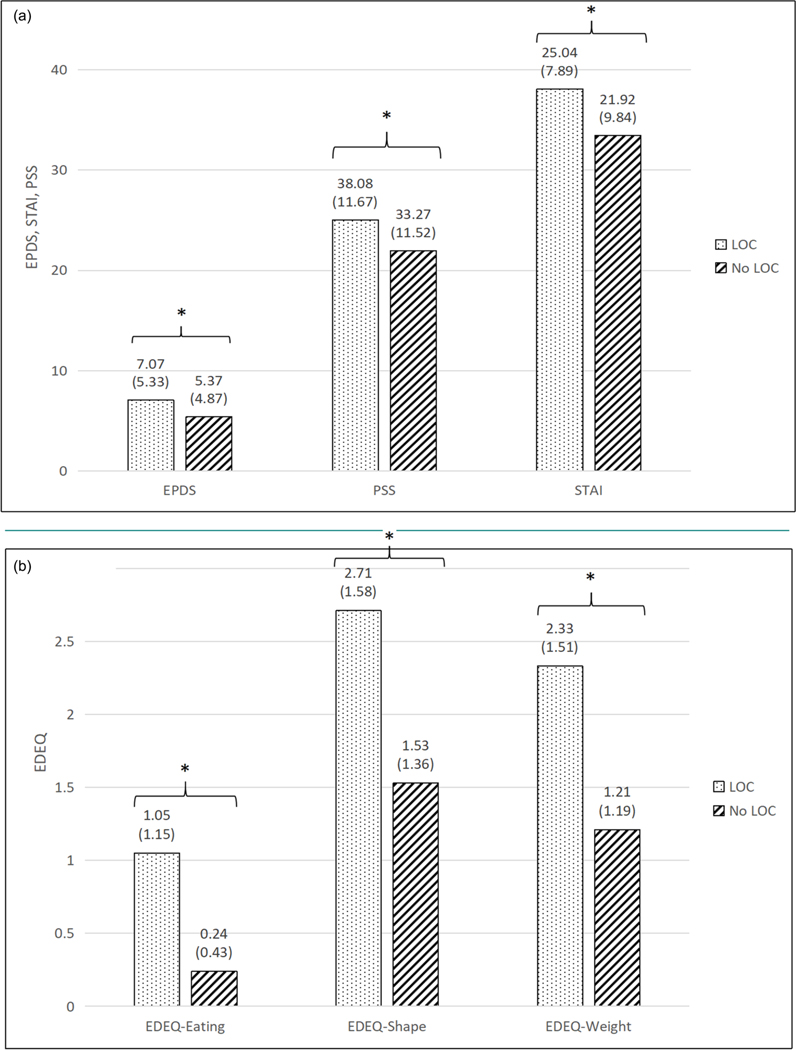
(a) Depressive symptoms (EPDS), perceived stress (PSS) and anxiety symptoms (STAI) by the presence or absence of prenatal LOC. (b) Levels of eating (EDEQ-eating) shape (EDEQ-shape) and weight (EDEQ-weight) concerns by presence or absence of prenatal LOC.

Pregnant individuals with prenatal LOC endorsed significantly more eating (1.1 ± 1.2 vs. 0.2 ± 0.4), shape (2.7 ± 1.6 vs. 1.5 ± 1.4), and weight (2.3 ± 1.5 vs. 1.2 ± 1.2*, p*s< .001) concerns than did those without prenatal LOC (See Figure 1). As expected, individuals who experienced more frequent LOC episodes had more severe eating (*β* = 0.15), shape (*β* = 0.17), and weight concerns (*β* = 0.16, *p*s<.001) than did those without prenatal LOC (See Table 2).

### Prenatal LOC and distress

Individuals with any prenatal LOC also endorsed more depressive (7.1 ± 0.3 vs 5.4 ± 4.9) and anxiety (38.1 ± 11.7 vs. 33.4 ± 11.7) symptoms and reported greater perceived stress (25.0 ± 7.9 vs. 22.0 ± 9.9, *p*s<.001*)*. Similarly, as shown in Table 2, LOC episode frequency was associated with greater psychological distress. Individuals who reported more frequent episodes of LOC also reported more symptoms of depression (*β* = 0.25, *p*=.003), anxiety (*β* = 0.70, *p*<.001) and perceived stress (*β* = 0.44, *p*=.005; See Table 2).

## Discussion

Approximately one-third of pregnant individuals with BMIs >25 enrolled in a perinatal health promotion trial reported prenatal LOC. This rate of LOC is consistent with rates of LOC in samples of both pregnant and non-pregnant individuals [6,7,16,17] As hypothesized, prenatal LOC was associated with greater concerns about eating, body shape, and weight and with higher levels of psychosocial distress. The link between LOC and symptoms of both disordered eating attitudes and psychological distress was robust, as evidenced by its presence even after accounting for factors that might increase an individual’s risk of experiencing LOC. Moreover, consistent with our hypotheses, the relationship between prenatal LOC and both disordered eating attitudes and psychological distress was linear. That is, pregnant individuals with more frequent episodes of LOC in the past month had greater concerns about body shape, body weight, and eating, endorsed more depressive and anxiety symptoms, and reported more perceived stress. Thus, the psychopathology correlates of prenatal LOC appear to be similar to those of LOC experienced outside of context of pregnancy.

Understanding the ways in which the experience of LOC during pregnancy relates to other markers of prenatal wellbeing is important in efforts to promote the short- and long-term health of perinatal people. Emerging research has begun to establish a link between prenatal disordered eating and postpartum physical and mental health. For example, individuals who report more prenatal psychological distress are at increased risk for postpartum depression and anxiety [40,41], and disordered eating attitudes during pregnancy may be linked to increased risk for continued postpartum disordered eating [42,43]. There is also a strong link between prenatal eating behavior and risk for later cardiovascular disease [23]. Given that prenatal LOC is associated with higher levels of disordered eating attitudes and psychological distress, it may be a particularly important treatment target to potentially improve mental and physical health during the perinatal period and beyond. Indeed, outside of pregnancy, data indicate that evidence-based treatments for disorders marked by LOC (e.g. binge eating disorder) result in improvements in disordered eating attitudes and psychological distress [44]. However, given that LOC treatments have been developed and tested exclusively outside of pregnancy [45], additional research is needed to understand how prenatal LOC responds to treatment and whether addressing prenatal LOC improves other eating disorder attitudes and general psychopathology

Notably, although it has become common to screen for depressive symptoms in prenatal settings [46], screening for disordered eating attitudes and behaviors is rare during prenatal care [34,47], despite evidence that LOC can develop in pregnancy [16]. Recently, several eating psychopathology measures, including brief versions, have been developed, adapted, and/or validated for use in pregnancy [48]. Given the prevalence of prenatal LOC and its contribution to psychological distress, screening for this eating disordered behavior is important during pregnancy. Moreover, additional work to refine and adapt instruments to assess a range of eating behavior and eating-related concerns during the prenatal period is merited [49].

These data are novel in documenting the unique effect of LOC on general and eating disorder psychopathology, even after accounting for propensity toward experiencing LOC. Although the use of propensity scores with additional covariate adjustment to model the effect of LOC on disordered eating attitudes and other markers of psychological distress helped to reduce overt bias associated with the cross-sectional nature of these data, the findings should be interpreted cautiously. Data from other samples suggest that shape and weight concerns increase from the middle of pregnancy through approximately two months postpartum in community samples [50]. In addition, little research has investigated whether prenatal LOC declines across pregnancy, although other aspects of disordered eating have been shown to improve across pregnancy [51]. One study conducted among individuals without disordered eating, with a current eating disorder, and with a history of an eating disorder, found that individuals reported more LOC episodes during the first or second trimesters than during the third trimester [52]. Further longitudinal research is needed to determine the relationships between LOC, which may be more common earlier in pregnancy [52], and disordered eating attitudes and other markers of psychological distress across the perinatal period as well as to examine the impact of of eating and other psychiatric disorders across the lifespan on psychological symptoms experienced during pregnancy. Moreover, the present study enrolled pregnant participants with a BMI ≥25, given that LOC has been found to be more common among individuals with a BMI ≥25. In addition, current gestational weight gain guidelines are based on pre-pregnancy BMI, and birthing individuals who begin pregnancy with a BMI ≥25 are more likely to exceed gestational weight gain guidelines. Thus, there are conceptual as well as practical considerations for focusing on individuals with a BMI ≥25 in this study. To increase generalizability across all pregnant people, it will be important to conduct future research among individuals across the weight spectrum to understand potential differences in LOC and associations with disordered eating and other mental health outcomes.

Despite these limitations, the finding that one-third of pregnant individuals with BMIs ≥ 25 endorsed LOC, and that those who report this behavior are also likely to report more concerns about body shape and weight and greater psychological distress, suggests the importance of addressing LOC during pregnancy. It is also noteworthy that prenatal LOC in this sample was assessed using a self-report questionnaire, and estimates were consistent with studies assessing prenatal LOC *via* interview [22], suggesting that LOC may be a behavioral factor that is easy to screen for during pregnancy. Screening for and addressing LOC during pregnancy may be important to improving health during pregnancy, postpartum, and across the lifespan.

## Figures and Tables

**Table 1.** Demographic characteristics.

Mean (SD)	Groups	All (*N* = 312)
Age (years)**		31.01 (4.91)
Gestational Length (weeks)**		13.61 (2.71)
Pre-pregnancy Weight (lbs)		191.41 (40.27)
Pre-pregnancy BMI (kg/m^2^)**		32.14 (6.23)
**n (%)**		
Race**	Asian	5 (1.60%)
	American Indian or Alaska Native	0 (0%)
	Black or African American	77 (24.68%)
	Multi-racial	15 (4.81%)
	Native Hawaiian or Other Pacific Islander	1 (0.32%)
	White	214 (68.59%)
Ethnicity * **	Hispanic or Latina	15 (4.8%)
Education**	>High school degree	265 (84.9%)
Employment**	Full-time/part-time for pay	234 (75.0%)
	Not working for pay /Leave of absence /Maternity leave /Other	78 (25%)
Income * **	>$30,000/year	214 (68.8%)
	≤$30,000/year	97 (31.2%)
Health Insurance * **	Yes	310 (99.7%)
In a Relationship**	Yes	284 (91.0%)
Previous/Current Smoker**	Yes	124 (39.7%)
Any LOC Days/Episodes	Yes	107 (34.3%)
Numbers of LOC Episodes		4.75 (4.52)

* *N* = 311.

** used in calculating propensity score.

Note: One individual was 17 years old (i.e. between 14–18 years).

**Table 2.** Relationships of LOC presence and episode frequency with psychological distress and eating disorder attitudes.

			LOC vs. No LOC		LOC Episode Frequency	
	LOC M (SD)	No LOC M (SD)	*t*-test (df)	*p* value	*β* coefficient (Standard Error)	*p* value
Depressive Symptoms	7.07 ± 5.33	5.40 ± 4.87	−2.70 (198.61)	0.007	0.26 (0.108)	0.016
Perceived Stress *(n = 311)*	25.04 ± 7.89	21.97 ± 9.87	−2.98 (259.88)	0.003	0.55 (0.203)	0.007
Anxiety *(n = 311)*	38.08 ± 11.67	33.43 ± 11.72	−3.32 (213.12)	0.001	0.84 (0.249)	0.001
Eating Concerns	1.05 ± 1.15	0.24 ± 0.44	−6.99 (122.39)	<0.001	0.20 (0.014)	<0.001
Shape Concerns	2.71 ± 1.58	1.53 ± 1.37	−6.50 (190.93)	<0.001	0.21 (0.030)	<0.001
Weight Concerns	2.33 ± 1.51	1.21 ± 1.19	−6.65 (176.12)	<0.001	0.20 (0.028)	<0.001

Note. Regression analyses controlled for propensity score.

## Data Availability

The data that support the findings of this study are available from the corresponding author, [MDL], upon reasonable request.
